# The complete chloroplast genome sequence of the *Sechium edule* (Jacq.) Swartz. (Cucurbitaceae)

**DOI:** 10.1080/23802359.2020.1847614

**Published:** 2021-01-12

**Authors:** Haonan Cui, Zicheng Zhu, Zhaokai Lu, Zhuo Ding, Chen Zhang, Feishi Luan

**Affiliations:** a College of Horticulture and Landscape Architecture, Northeast Agricultural University, Harbin, PR China; b Key Laboratory of Biology and Genetic Improvement of Horticulture Crops (Northeast Region), Ministry of Agriculture and Rural Affairs, Harbin, PR China

**Keywords:** *Sechium edule*, chloroplast genome, phylogenetic analysis

## Abstract

*Sechium edule* (Jacq.) Swartz is an important vegetable with both food and medicinal values. The complete chloroplast genome sequence of *S. edule* has been reported in this study. The total genome size is 154,558 bp in length and contains a pair of inverted repeats (IRs) of 19,128 bp, which were separated by large single-copy (LSC) and small single-copy (SSC) of 98,806 and 17,496 bp, respectively. A total of 122 genes were predicted including 78 protein-coding genes, 8 rRNA genes, and 36 tRNA genes. Further, the phylogenetic analysis confirmed that *S. edule* belongs to the family Cucurbitaceae. The complete chloroplast genome of *S. edule* would play a significant role in the development of molecular markers for plant phylogenetic and population genetic studies.

*Sechium edule* (Jacq.) Swartz (chayote) is a perennial herbaceous climber in the Cucurbitaceae family, with tendrils and tuberous roots, cultivated in Mexico since pre-Columbian times. The edible fruit is popularly known as many names. The main producer countries of *S. edule* include Mexico, Costa Rica, Brazil, and the Dominican Republic. This fruit is mainly consumed using traditional cooking methods (Vieira et al. 2019). In addition to its nutritional value, different parts of this plant are also used in traditional medicine to treat kidney disease and other ailments such as diabetes, obesity, and arteriosclerosis (Castro Rodríguez et al. 2015; Diaz-de-Cerio et al. 2019). The chloroplast genome is an important molecular tool for the taxonomic identification of plants (Techen et al. 2014). The aim of this study was sequencing the complete chloroplast genome of *S. edule* to facilitate species identification, germplasm exploration, and phylogenetic relationships and providing a basis for evolutionary analysis of Cucurbitaceae.

The sample of *S. edule* (Voucher specimen: FSG01) was stored in the Laboratory of Molecular Genetic Breeding of Watermelon and Melon at Northeast Agricultural University (45°44′23.8′′N, 126°43′16.7′′E), Harbin, China. The total genomic DNA of *S. edule* was extracted from leaf tissues with the modified CTAB method (Montero-Pau et al. 2018). Genomic DNA was subjected to construct a 500 bp pair-end library and sequenced by Illumina HiSeq 2500. After sequencing and base quality control, a total of 12.48 Gb of sequence data in fastq format was obtained. The draft genome sequence was assembled by NOVOPlasty version 4.0 (Dierckxsens et al. 2017). The assembled genome was annotated CPGAVAS2 (Linchun et al. 2019) and the annotation was corrected with Geneious version 11.0.3 (Kearse et al. 2012).

The complete chloroplast genome of *S. edule* is 154,558 bp and exhibits a typical quadripartite structure, consisting of a pair of inverted repeat regions (IRs, 19,128 bp) separated by the large single-copy (LSC, 98,806 bp) and small single-copy (SSC, 17,496 bp) regions. There is a total of 122 genes, including 78 protein-coding genes, 8 rRNA genes, and 36 tRNA genes. The overall GC content of the chloroplast genome was 37.20%.

To determine the phylogenetic position of *S. edule*, a phylogenetic analysis was conducted with 14 complete chloroplast genomes, 13 of these belonged to Cucurbitaceae and one to *Vitis vinifera* which is considered an outgroup. The phylogenetic tree was constructed by maximum likelihood method using the program MAFFT version 7.407 (Nakamura et al. 2018) and MEGA version 10.0.4 (Kumar et al. 2018) with bootstrap set to 1000. The results showed that *S. edule* was clustered into the family Cucurbitaceae, and other species in the different families were also well clustered into their corresponding clades (Figure 1).

**Figure 1.**
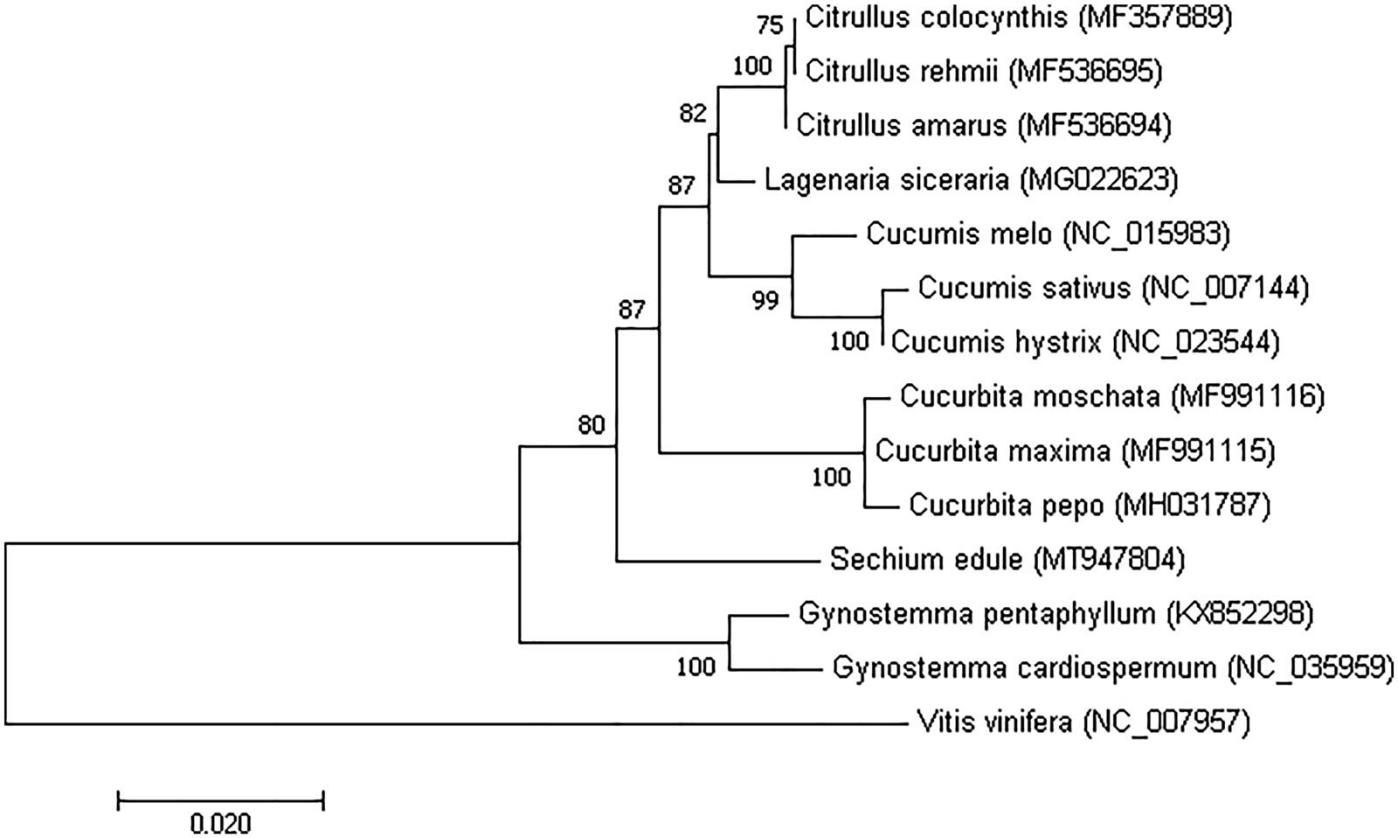
Phylogenetic tree showing the relationship between *S. edule* and other 12 species belonging to Cucurbitaceae. The phylogenetic tree was constructed based on the complete chloroplast genomes using maximum likelihood (ML) with 1000 bootstrap replicates. Numbers in each node indicated the bootstrap support values.

## Data Availability

The data that support the findings of this study are openly available in NCBI at https://www.ncbi.nlm.nih.gov/nuccore/MT947804, the resequencing data were deposited in GenBank (https://www.ncbi.nlm.nih.gov/) under BioProject ID PRJNA667136, or available from the corresponding author.
